# Nanosecond Pulsed Electric Fields (nsPEFs) Regulate Phenotypes of Chondrocytes through Wnt/β-catenin Signaling Pathway

**DOI:** 10.1038/srep05836

**Published:** 2014-07-25

**Authors:** Kun Zhang, Jinsong Guo, Zigang Ge, Jue Zhang

**Affiliations:** 1 Department of Biomedical Engineering, College of Engineering, Peking University, Beijing 100871, China; 2 Institute of Biomechaics and Biomedical Engineering, College of Engineering, Peking University, Beijing 100871, China; 3 Center for Biomedical Materials and Tissue Engineering, Academy for Advanced Interdisciplinary Studies, Peking University, Beijing 100871, China; 4 Arthritis Clinic and Research Center, Peking University People's Hospital, Beijing, 100871, China; 5 Center for BioMed-X Research, Academy for Advanced Interdisciplinary Studies, Peking University, Beijing 100871, China

## Abstract

Nanosecond pulsed electric fields (nsPEFs) characterized by high voltage, low energy and non-thermal effects, have been broadly investigated as a potential tumor therapy; however, little is known about their effects on somatic cells. In this current study, we evaluated effects of nsPEFs on the phenotype of chondrocytes (morphology, glycosaminoglycan (GAG) content, proliferation and gene expression) and explored the mechanisms involved. Our results demonstrated that exposing chondrocytes to nsPEFs led to enhanced proliferation and dedifferentiation, evidenced by the upregulated gene expression of collagen type I (COL I) and downregulated gene expression of Sox9, collagen type II (COL II) and aggrecan (AGG) with activation of the wnt/β-catenin signaling pathway. Inhibition of the wnt/β-catenin pathway partially blocked these effects. Thus we concluded that nsPEFs induce dedifferentiation of chondrocytes partially through transient activation of the wnt/β-catenin signaling pathway.

Millisecond or microsecond pulsed electric fields (PEFs) have been shown to facilitate delivery of drugs and transfer of genes into cells^1^,^2^. PEFs induce a transient transmembrane potential of approximately 200 mV–1 V with limited thermal effects^1^,^3^. Nanosecond pulsed electric fields (nsPEFs) have shown some advantages over millisecond or microsecond PEFs, as they can achieve similar membrane potentials with higher voltage differentials, lower energy and negligible thermal effects^4^. nsPEFs generate nanopores in cell membranes smaller than those induced by traditional PEFs, which only allow transfer of small molecules such as water, chloride ions and alkali metal cations^5^. Furthermore, nsPEFs allow for selective and operable cell electrofusion independent of the size of the cell^6^. In addition to changes in the cell membrane, nsPEFs also lead to a series of changes that occur subsequently within the cells, such as transfer of phosphatidylserine from the interior to the exterior of lipid membranes^7^, sparkler morphology of intracellular granules with cytoplasmic free calcein staining^8^, release of cellular calcium ions from the endoplasmic reticulum^9^, and activation of mitogen-activated protein kinases (MAPK) pathways^10^. Finally, nsPEFs induce tumor cell apoptosis both *in vivo* and *in vitro* through release of cytochrome *c*^11^,^12^. Although the function of nsPEFs has been explored in multiple cell lines, including T-lymphocytes cell lines, hematologic cell lines and pancreatic cancer cell lines^11^,^13^, limited research has been conducted on primary mammalian somatic cells.

Chondrocytes are critical for maintenance and regeneration of cartilage, and several signaling pathways regulate phenotypes of differentiation, hypertrophy, proliferation, and dedifferentiation. Transforming growth factor-beta (TGF-β) has been shown to promote the differentiation of chondrocyte^14^. Bone morphogenetic proteins (BMPs) stimulate chondrocyte maturation, while parathyroid hormone (PTH) and parathyroid hormone-related peptide (PTHrP) inhibit the rate of maturation^15^,^16^. The wnt/β-catenin pathway causes dedifferentiation of chondrocytes, and promotes cell proliferation^17^,^18^. The phenotypes of chondrocytes are tightly regulated by physical factors, including electrical stimuli^19^. Traditional PEFs have shown to increase protein synthesis and subsequently promote the healing response in osteochondral defects through upregulation of aggrecan (AGG) and collagen type II (COL II) in chondrocytes^20^,^21^,^22^. Low voltage (less than 100 mV/cm) treatments were used in the aforementioned research due to the thermal effects of PEFs. Although the biological effects induced by nsPEFs are largely unknown, it has been reported that MAPK pathways, general control non-depressible-2 (GCN2) pathways and double-stranded RNA-dependent protein kinase-like endoplasmic reticulum kinase (PERK) pathways are involved^10^,^23^.

In this study, we evaluated the phenotypic effects of nsPEFs on chondrocytes and found that nsPEFs enhanced cell proliferation while causing dedifferentiation by upregulating gene expression of type I collagen (COL I) and downregulating gene expression of COL II. We then explored whether activation of the wnt/β-catenin signaling pathway was involved in these phenotypic changes (Fig. 1).

## Results

### nsPEFs enhance proliferation of chondrocytes

**Figure 2:**
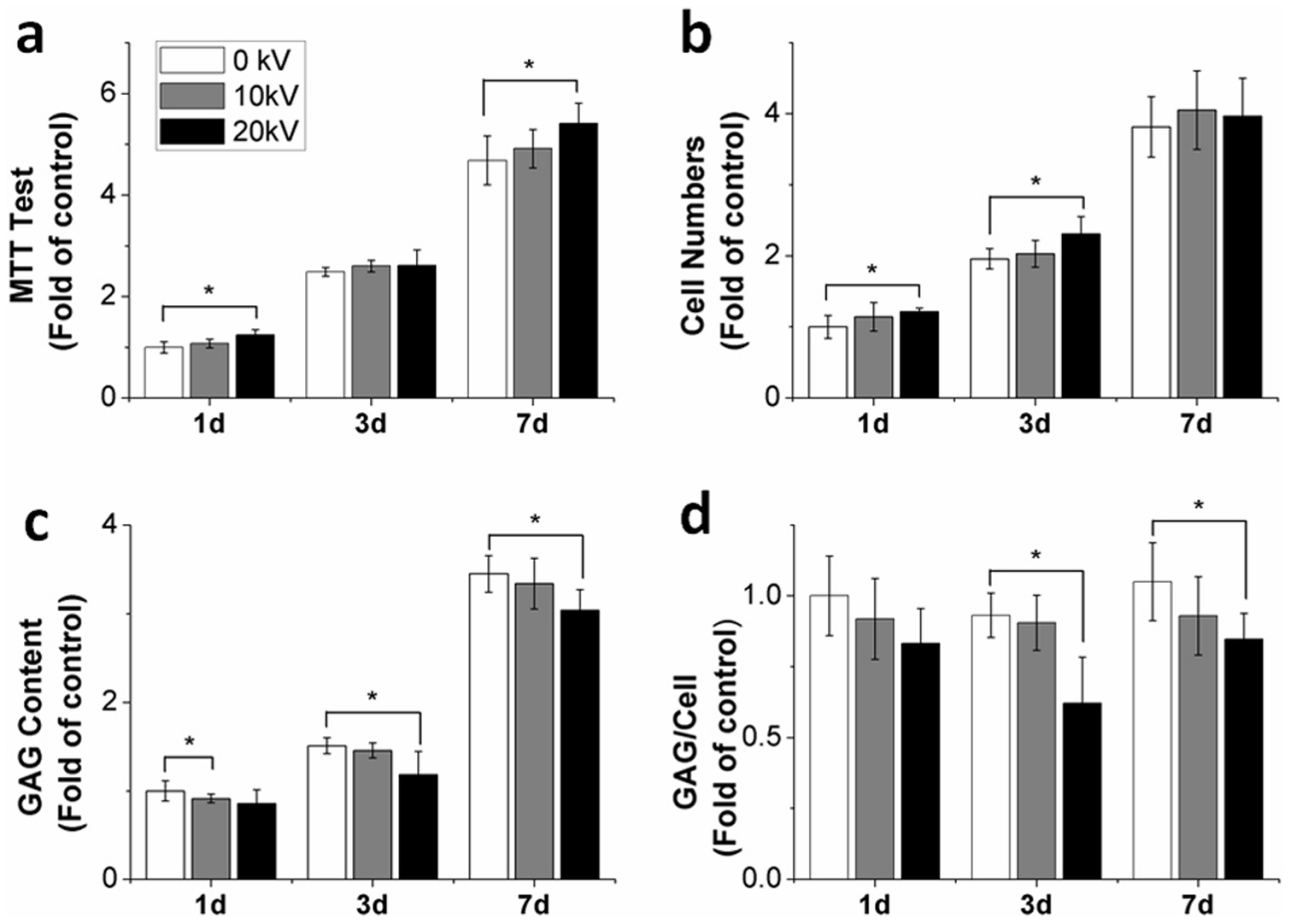
Induction of dedifferentiated phenotype of chondrocytes after nsPEF treatment. (a) The toxicity of nsPEFs at 10 kV/cm and 20 kV/cm was evaluated at days 1, 3 and 7. nsPEFs had a nontoxic effect on chondrocytes (n = 5). (b) Cell proliferation was evaluated at days 1, 3 and 7 (n = 5). Cell proliferation increased after nsPEF treatment. (c) GAG content was evaluated at days 1, 3 and 7 (n = 4). GAG content decreased after nsPEF treatment. (d) GAG content produced per chondrocyte was evaluated by the total GAG content divided by cell numbers; it was repressed after 10 kV/cm and 20 kV/cm nsPEF treatment (n = 4). Data expressed as mean ± s.d. * = p < 0.05.

Based on favorable results obtained in previous reports and our pilot study, 5 pulses of 100 ns nsPEFs at 10 or 20 kV/cm were used in this current study^10^. Cytotoxicity of nsPEFs on chondrocytes was evaluated with 3-(4,5-Dimethyl-2-thiazolyl)-2,5-diphenyl-2H-tetrazolium bromide (MTT) assay^24^. Exposing chondrocytes to nsPEFs at 10 kV/cm increased absorbance values obtained from MTT assay to 1.07-fold, 1.05-fold and 1.05-fold, while nsPEFs at 20 kV/cm caused a 1.24-fold (p = 0.002), 1.04-fold and 1.16-fold (p = 0.018) increase at days 1, 3 and 7, respectively (Fig. 2a). nsPEFs appeared to have no significant effect on chondrocyte morphology (Supplementary Fig. S1). nsPEFs at 10 kV/cm revealed a slight increase in proliferation of chondrocytes at day 1 (1.14-fold), day 3 (1.03-fold) and day 7 (1.06-fold), whereas nsPEFs at 20 kV/cm significantly increased cell proliferation at day 1 (1.21-fold, p = 0.02), day 3 (1.18-fold, p = 0.02) and day 7 (1.04-fold) (Fig. 2b).

### nsPEFs downregulate glycosaminoglycan (GAG) production

Results obtained demonstrated that nsPEFs at 10 kV/cm decreased GAG production at day 1 (0.91-fold), day 3 (0.96-fold) and day 7 (0.97-fold). Moreover, nsPEFs at 20 kV/cm also further decreased GAG production at day 1 (0.86-fold) and significantly at day 3 (0.78-fold, p = 0.046) and day 7 (0.88-fold, p = 0.041) (Fig. 2c). In addition, nsPEFs at 10 kV/cm decreased the GAG/cell ratio to 0.92-fold, 0.97-fold and 0.88-fold at days 1, 3 and 7, while nsPEFs at 20 kV/cm decreased the GAG/cell ratio at day 1 (0.83-fold), with significant findings at day 3 (0.67-fold, p = 0.012) and day 7 (0.81-fold, p = 0.047) (Fig. 2d).

### nsPEFs downregulate expression of functional genes

**Figure 3:**
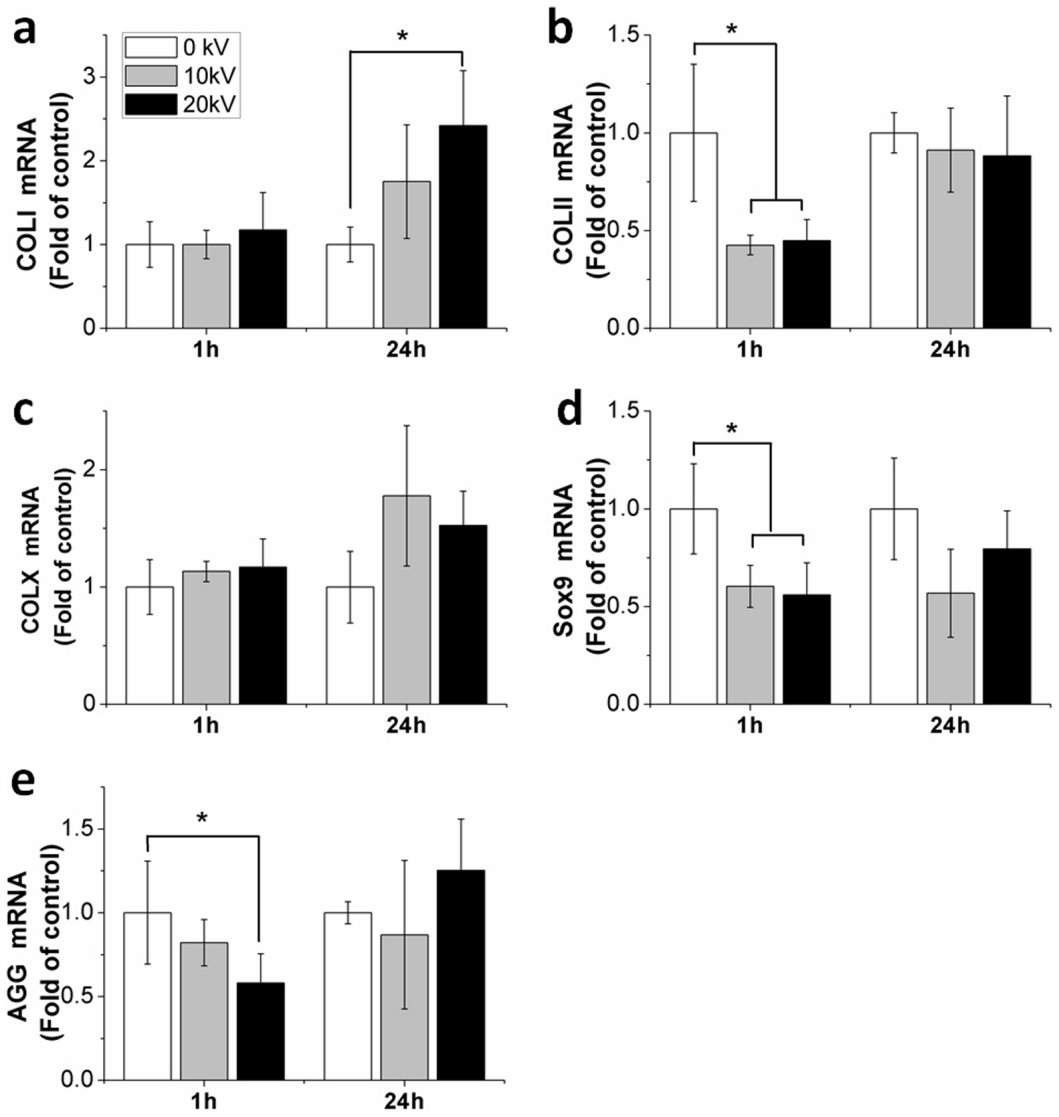
Functional gene expression of chondrocytes after nsPEF treatment. Gene expression was analyzed at 1 hour for immediate effects and following a recovery period of 24 hours after 10 kV/cm and 20 kV/cm nsPEF treatment, respectively. (a) COL I; (b) COL II; (c) COL X; (d) Sox9; (e) AGG. Quantitative real-time PCR analysis was performed (n = 3). Untreated chondrocytes (0 kV) served as controls. Data expressed as mean ± s.d. * = p < 0.05.

Effects of nsPEFs on gene expression were evaluated at 1 hour and 24 hours after nsPEF treatment. At 1 hour, nsPEFs at 10 kV/cm decreased gene expression of COL II significantly to 0.43-fold (p = 0.017), while nsPEFs at 20 kV/cm decreased COL II gene expression to 0.45-fold (p = 0.02) (Fig. 3b). Gene expression of Sox9 was significantly downregulated to 0.60-fold (p = 0.026) at 10 kV/cm and 0.56-fold (p = 0.014) at 20 kV/cm (Fig. 3d). Similarly, decreased induction of AGG was subsequently detected at 0.82-fold and 0.58-fold (p = 0.047) after 10 kV/cm and 20 kV/cm nsPEF treatment (Fig. 3e). Interestingly, nsPEFs increased gene expression of COL I to 1.02-fold at 10 kV/cm and 1.2-fold at 20 kV/cm. An increase in COL X gene expression to 1.13-fold at 10 kV/cm and 1.17-fold at 20 kV/cm (Fig. 3a, c) was also observed.

At 24 hours after nsPEF treatment, gene expression showed an increase compared to the gene expression at 1 hour. An additive effect of COL I was indicated by an increase in the gene expression to 1.75-fold at 10 kV/cm and 2.42-fold (p = 0.02) at 20 kV/cm. Gene expression of COL II, COL X, Sox9 and AGG of nsPEF-altered returned to the levels of untreated chondrocytes, and no significant difference was observed when compared to the untreated cells.

### nsPEFs activate wnt/β-catenin signaling pathway

**Figure 4:**
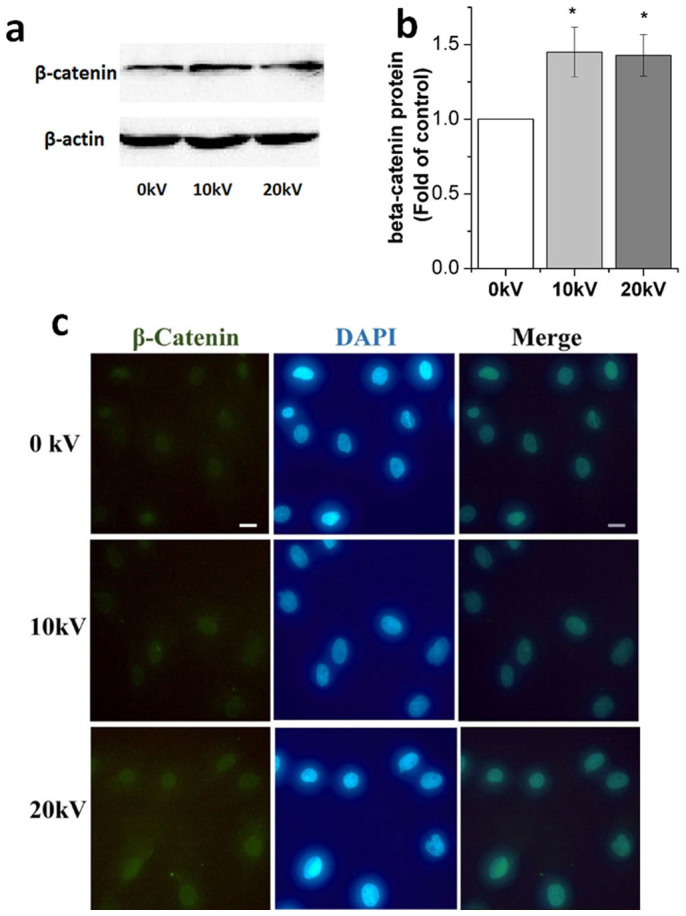
Activated wnt/β-catenin signaling pathway after nsPEF treatment. (a) β-catenin protein expression performed with western blotting analysis using specific antibodies for β-catenin at 1 hour after 0, 10 and 20 kV/cm nsPEF treatment. (b) Histogram of quantitative analysis of relative β-catenin expression. (c) Immunofluorescence images of chondrocytes after 0, 10 and 20 kV/cm nsPEF treatment. The β-catenin is labeled with specific antibodies (green). Nucleus is stained with DAPI (blue). Scale bar represents 10 μm. Data expressed as mean ± s.d. * = p < 0.05.

**Figure 5:**
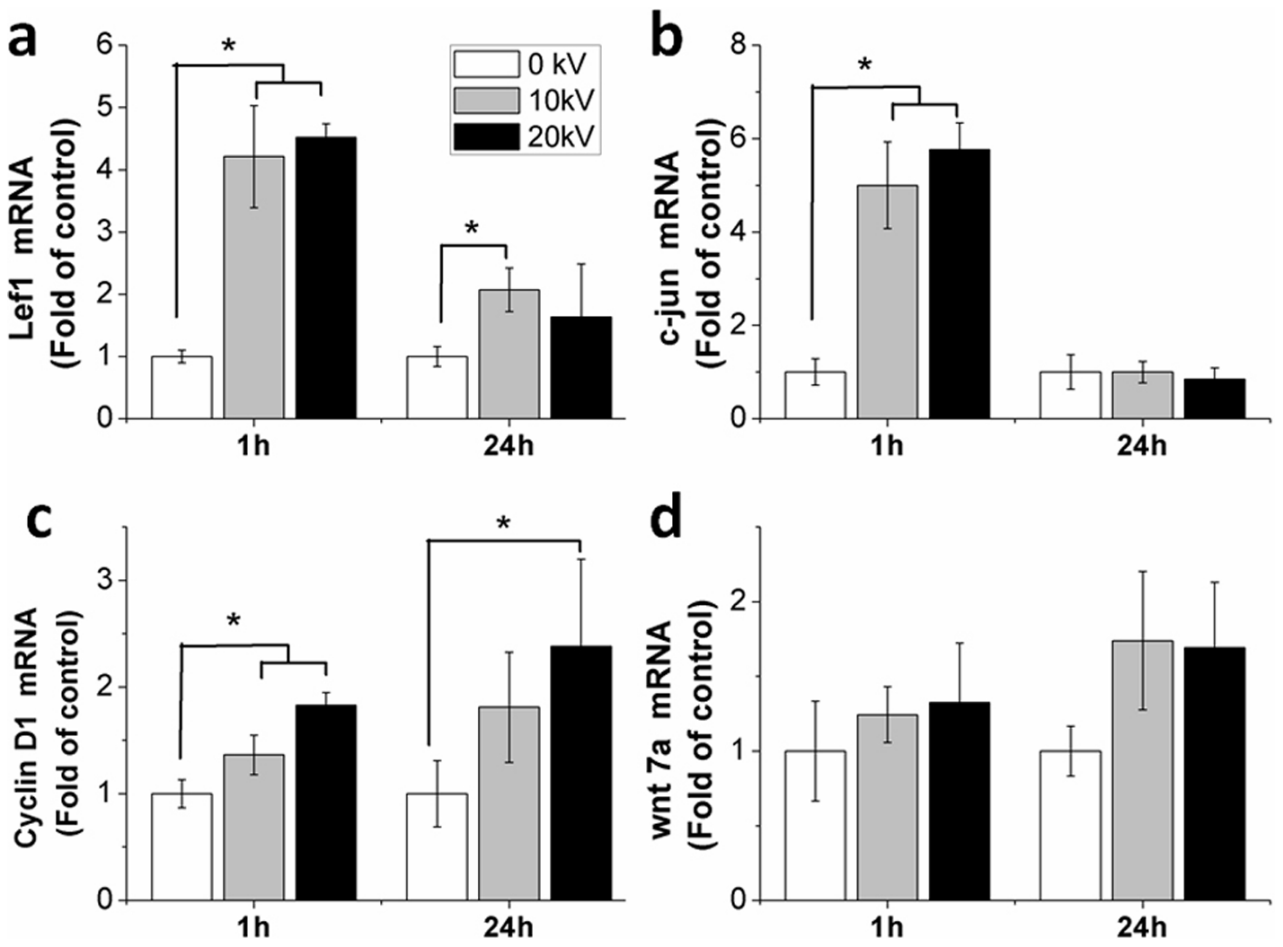
Influence of nsPEFs on gene expression related to wnt/β-catenin signaling. Gene expression was detected immediately at 1 hour and following a 24 hour recovery after 0, 10 and 20 kV/cm nsPEF treatment, respectively. (a) Lef1; (b) c-jun; (c) cyclin D1; (d) wnt7a. Quantitative real-time PCR analysis was performed (n = 3). Untreated chondrocytes (0 kV) served as controls. Data expressed as mean ± s.d. * = p < 0.05.

Expression of β-catenin protein increased significantly by 45% (p = 0.005) and 42% (p = 0.006) 1 hour after 10 kV/cm and 20 kV/cm nsPEF treatment, respectively (Fig. 4a, b). As shown, β-catenin accumulated in the nucleus of the cells after nsPEF treatment (Fig. 4c). 10 kV/cm and 20 kV/cm nsPEFs increased the wnt7a gene expression to 1.2-fold and 1.3-fold (Fig. 5d), respectively. Downstream genes of the wnt/β-catenin signaling pathway; Lef1, c-jun and cyclin D1, were also evaluated. Gene expression of Lef1 significantly increased to 4.2-fold (p = 0.001) and 4.5-fold (p = 0.001) after 10 kV/cm and 20 kV/cm nsPEF treatment (Fig. 5a), c-jun increased to 5.1-fold (p = 0.001) at 10 kV/cm and 5.9-fold (p = 0.001) at 20 kV/cm (Fig. 5b) and gene expression of cyclin D1 increased to 1.4-fold (p = 0.04) at 10 kV/cm and 1.8-fold (p = 0.01) at 20 kV/cm (Fig. 5c).

Our findings demonstrated that after 24 hours, nsPEFs induced a decrease in Lef1 and c-jun gene expression in comparison to the gene expression observed after 1 hour. nsPEFs increased gene expression of Lef1 to 2.1-fold (p = 0.048) at 10 kV/cm and cyclin D1 to 2.4-fold (p = 0.027) at 20 kV/cm. No significant differences in the gene expression levels of other genes were observed when compared to the untreated cells.

### Inhibition of wnt/β-catenin pathway partially blocks effects of nsPEFs

**Figure 6:**
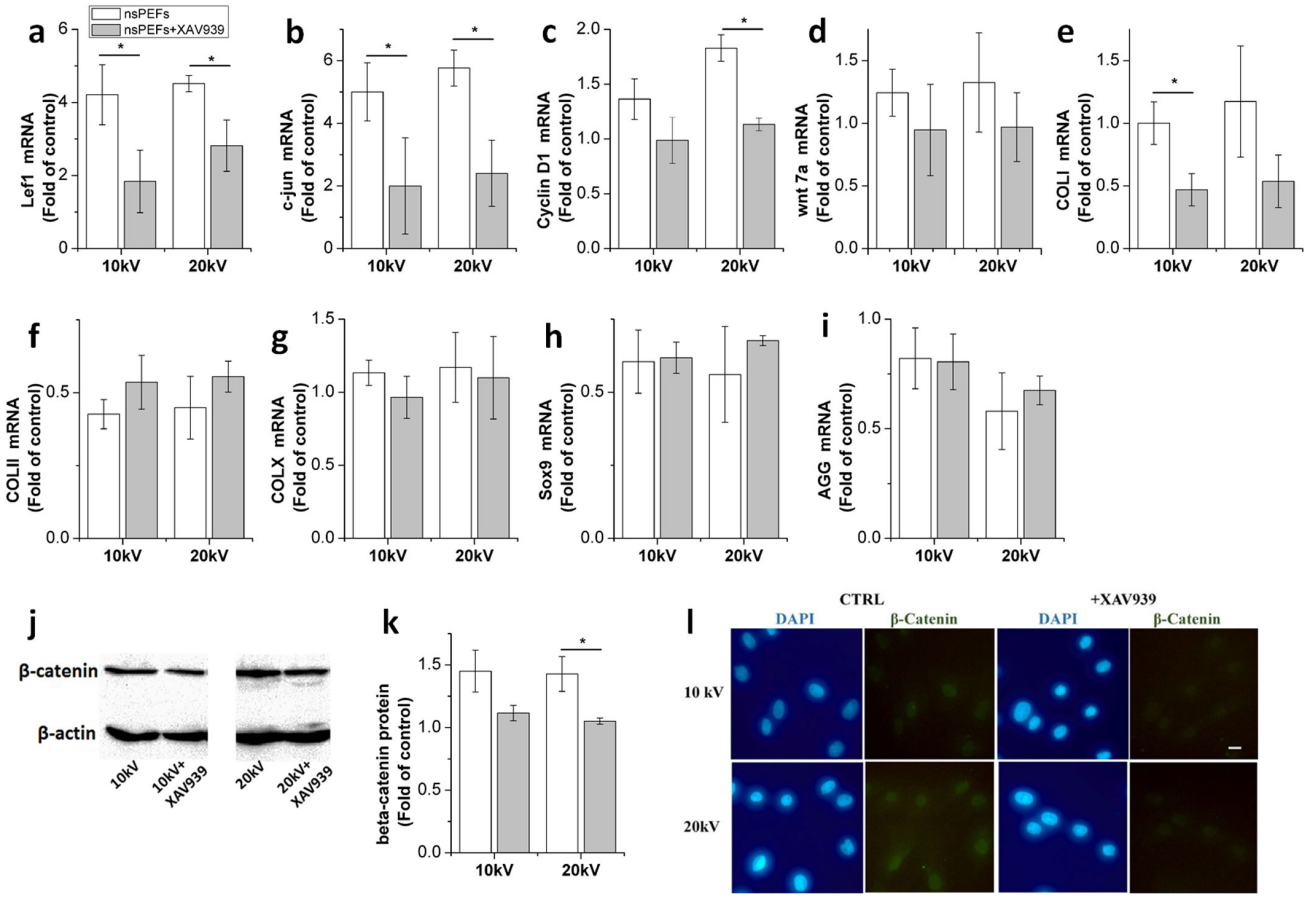
Influence of inhibition of wnt/β-catenin signaling on gene and protein expression after nsPEF treatment. Gene expression was detected at 1 hour after 0, 10 and 20 kV/cm nsPEF treatment in the presence or absence of XAV939 (wnt inhibitor) compared with untreated chondrocytes. (a) Lef1; (b) c-jun; (c) cyclin D1; (d) wnt7a; (e) COL I; (f) COL II; (g) COL X; (h) Sox9; (i) AGG. Quantitative real-time PCR analysis was performed (n = 3). Untreated chondrocytes (0 kV) served as controls. (j) β-catenin protein expression was measured in the presence or absence of XAV939 with application of nsPEF treatment. (k) Quantitative analysis of β-catenin expression. (l) Immunofluorescence images of chondrocytes after nsPEF co-treatment with or without XAV939. The β-catenin is labeled with specific antibodies (green). Nucleus is stained with DAPI (blue). Scale bar represents 10 μm. Data expressed as mean ± s.d. * = p < 0.05.

Incubation with XAV939 reverted nsPEF-induced upregulation of β-catenin (Fig. 6j–6l), wnt7a gene (Fig. 6d) and the downstream genes of wnt, Lef1 (Fig. 6a), c-jun (Fig. 6b) and cyclin D1 (Fig. 6c) by inhibition ranging from 30% to 60%. When the effects of inhibition were compared with the untreated chondrocytes, β-catenin, cyclin D1 and wnt7a levels were found to have decreased back to normal levels. Although Lef1 and c-jun levels recorded were higher after inhibition than those in the control group, no significant difference was observed. Results indicated that the wnt/β-catenin pathway played an integral role as a functional pathway after nsPEF treatment of chondrocytes. Functional gene expressions of COL II (Fig. 6f), Sox9 (Fig. 6h) and AGG (Fig. 6i) were elevated by approximately 20%, while COL I (Fig. 6e) was reduced by approximately 50% after XAV939 incubation with nsPEFs. Compared with the control group, functional gene expressions after inhibition were far less than those of untreated chondrocytes.

## Discussion

nsPEFs have profound effects on multiple organelles of a cell. nsPEFs generate large transmembrane potentials across cellular organelles with limited thermal effects. The most significant characteristic of nsPEFs is their short duration, which is less than the charging time of the plasma membrane with a microsecond range^2^,^25^,^26^. At high pulse duration, the outer plasma membrane with its large capacitance is markedly affected, and the potential across the interior is small. As pulse duration decreases, the outer membrane is likely shorted, and the applied voltage appears mainly across the interior organelles of the cell^27^. nsPEFs modify the potential and phosphatidylserine externalization of plasma membranes^7^, induce calcium ion bursts from the endoplasmic reticulum and cytochrome *c* release from the mitochondria^9^,^11^,^12^. Subsequently, nsPEFs influence nuclear activities by producing DNA speckles and RNA–protein complexes^28^,^29^, and produce newly-formed species of oxide, such as H_2_O_2_^30^. nsPEFs provide a potential way for cellular interaction in the absence of ligand or receptor by induction of nanopores^29^,^31^.

Effects of nsPEFs are usually transient. Typically, the nanopores can be formed within 5 nanoseconds after nsPEF treatment^7^. Calcium ions efflux from the endoplasmic reticulum within 10 seconds following nsPEF treatment^32^. nsPEFs induce a cell fusion process within 4 minutes^6^, and phosphorylate the cellular stress factor eIF2α with a peak at 1 hour post-treatment^23^. Our study showed, via a time course analysis, that nsPEFs caused rapid effects on gene expression. We monitored the gene expressions of COL II and c-jun mRNA changes at 0.5, 1, 2, 6 and 24 hours. COL II gene expression was found to undergo a rapid and statistically significant decrease after nsPEF treatment, while no significant difference was observed after a recovery period of 1 day (Supplementary Fig. S2a). C-jun gene expression increased immediately to a maximum level at 1 hour, and decreased to the normal level after a recovery period of 1 day (Supplementary Fig. S2b).

Fine-tuned activation of the wnt/β-catenin signaling pathway is essential to regulate the fate of chondrocytes in cartilage. The wnt/β-catenin pathway is involved in dedifferentiation of cultured chondrocytes^33^. Once activated, wnt ligands combine with frizzled receptors and coreceptors, such as lipoprotein receptors, to facilitate β-catenin accumulation, which subsequently enters the nucleus to regulate the transcription of genes, such as Lef1, c-jun and cyclin D1^34^. Activation of the wnt/β-catenin signaling leads to proliferation of chondrocytes via upregulation of cyclin D1, and dedifferentiation via Lef1, c-jun and Sox9^34^,^35^,^36^. Previous studies have illustrated that nsPEFs enhance transcription machinery of the nucleus, mediated through the penetration of intracellular membranes and the induction of nanopores^32^,^37^. In addition, Sox9 directly regulates COL II expression by binding the first intron sequences of COL II^38^, as well as AGG expression^39^. Although abnormal activation of wnt/β-catenin signaling leads to degradation of cartilage matrix and enhances dedifferentiation of chondrocytes^40^,^41^, inhibition of the wnt/β-catenin signaling also leads to apoptosis of chondrocytes in articular cartilage^42^. nsPEFs induce a transient response of the wnt/β-catenin pathway and can be used to regulate cells in a dose and time-dependent manner^43^.

The fact that inhibition of the wnt/β-catenin signaling can only partially block the effects of nsPEFs on chondrocytes hints that there are other mechanisms involved. The functional crosstalk between signaling pathways introduces the possibility that nsPEFs simultaneously activate multiple pathways. A previous study showed that nsPEF-activated gene expression which was inhibited by 30% after co-treatment with a JNK inhibitor, however gene expression was found to be 5-fold higher than the cells without nsPEF treatment^44^. nsPEFs activate and induce biochemical changes, such as bursts of calcium ions^45^,^26^. Calcium ions serve as second messengers and provide important upstream signals for cellular mechanisms to occur such as proliferation, differentiation and apoptosis^46^. Similar to previous reports, we found calcium ion release after nsPEF treatment (Supplementary Fig. S3). Although a calcium ion chelator effectively blocked calcium efflux, the chelator showed no effect on chondrocyte phenotypes (Supplementary Fig. S4). Previous research has shown that nanopores generated by nsPEF treatment facilitate bursts of calcium ion release, while traditional PEFs cause calcium ion release by altering the transmembrane energy barriers^7^. nsPEFs also influence nuclear activities and processes. Since DNA is heavily charged due to its folded spiral structure, it is sensitive to nsPEFs and forms speckles after nsPEF treatment^28^. nsPEFs induce small nuclear ribonucleoprotein particles and RNA-protein complexes, which are important in messenger RNA transcriptional functions^29^. nsPEFs affect cellular behavior by introducing a mechanical stress through thermoelastic expansion^23^,^47^. Therefore, the potential electrochemical influence of electrolysis in high voltage electric fields need to be considered^48^. Several parameters, such as strength, pulse duration and number of stimulating electric fields, also add complexity to the effects of nsPEFs^45^. Although the relationship between nsPEFs and cells is not lucid, nsPEFs remain a promising application, as they can potentially alter biochemical, biophysical and electrochemical properties of cells, with tunable parameters.

nsPEFs may have different effects on suspended and attached cells *in vitro*. Cells in a suspended state were subjected to nsPEFs within electric cuvettes, which provided an instantaneous and consistent distribution of an electric-field^49^. Similar trends of gene expression were observed in chondrocytes cultured in both an attached and a suspended state. The gene expressions of Lef1, c-jun, cyclin D1, wnt7a and COL I were upregulated, while the expression of COL II, Sox9 and AGG were downregulated (Supplementary Fig. S5). However, the change in gene expression of attached chondrocytes was less than that of suspended chondrocytes, and significant differences could be found in Lef1, c-jun, COLII and Sox9 expression. Results showed that chondrocytes cultured in the attached state were less expressive in comparison with those cultured in a suspended state. Culturing state is an important factor affecting cell physiological function since mechanical properties mainly depend on cellular cytoskeletal structure^50^. A previous report has shown that cell survival and genotoxic effects of non-adherent cells may be more sensitive than that of adherent cells after nsPEF treatment^28^. The cytoskeleton, a possible factor affecting cellular viability in both non-adherent and adherent cells, has been demonstrated to be less effective in sustaining cellular viability after nsPEF treatment^51^. In order to evaluate the cells in an adherent state, a standard culturing system needs to be developed, comparable to the electric cuvette system in the suspended state. One such possibility may be the development of a real-time visual microfluidic system for monitoring adherent cells combined with nsPEFs^52^.

As nsPEFs do not exist in a natural cellular environment, a comprehensive understanding of nsPEFs as well as the effects of superficial stimuli need to be further explored to determine any difference from previously known cellular functions^53^. Varied parameters of nsPEFs, such as duration, number of pulses and intensity, may be used to provide versatile tools to regulate different biological processes in the future. The effects of nsPEFs may depend on multiple parameters, such as depth, dose, cell type, cell attachment and tissue type. Understanding nsPEFs influence matrix metabolism and cross-talk signaling pathways may lead to potential methods to regulate genetics. Further research is needed to determine the applicability of nsPEFs on whole tissues.

## Methods

### Cell culture

Porcine articular cartilage tissue was cut into small pieces by a lancet, and washed with phosphate-buffered saline (PBS). The tissue pieces were collected and digested in 0.1% Collagenase II (17101-015, Gibico) dissolved in Dulbecco's modified Eagle's medium (DMEM, 31600-034, Invitrogen) at 37°C overnight. The isolated chondrocytes were harvested and cultured in monolayer in culture plates with the medium containing 90% DMEM, 10% fetal bovine serum (FBS, SV30087.02, Gibico), and 0.1% penicillin/streptomycin (PS) at 37°C in humidified atmosphere with 5% CO_2_. Cultured medium was changed every three days. When chondrocytes reached 85% confluency, they were trypsinized with 0.25% trypsin (27250-018, Invitrogen) and frozen in culture medium containing 10% dimethyl sulfoxide (DMSO, Merck). The thawed chondrocytes were prepared for further experimentation. To inhibit the effects of wnt activation, XAV939 (13596, 1 μM, Cayman Chemical) was utilized to pre-treat chondrocytes overnight before nsPEF treatment.

### Application of nsPEFs

The nsPEF generator was applied as previously described^54^. Digital phosphor oscilloscope (DPO4054, Tektronix) with a probe (P6015A, Tektronix) was utilized to monitor the voltage waveform. Chondrocytes were counted with a hemocytometer, and 1.0 × 10^6^ chondrocytes suspended in 500 μL culture medium were added to 0.2 cm gap cuvettes (Biosmith, aluminum plate electrodes, San Diego, CA). The experimental cuvettes were treated with 5 pulses of nsPEFs with 100 ns durations at 10 kV/cm or 20 kV/cm electric fields. Time between each pulse was about 1 s. Cuvettes that did not undergo nsPEF treatment served as the control group.

### Celltoxicity

The toxicity of nsPEFs was evaluated by 3-(4,5-Dimethyl-2-thiazolyl)-2,5-diphenyl-2H-tetrazolium bromide (MTT, M2128, Sigma) at days 1, 3 and 7. 5.0 × 10^3^ chondrocytes/well were seeded in 96-well plates. 20 mL of 5 mg/mL MTT solution was added to each well, which were then incubated at 37°C for 3 hours. The solution was emptied and 150 μL of DMSO was added. Optical density was measured at a wavelength of 570 nm with Microplate Reader (680, Bio-rad) to determine viable chondrocytes. The value was expressed as the ratio of the experimental group divided by the control group. Five samples of each group were measured.

### Cell proliferation

Cell proliferation was assayed with Hoechst 33258 (H6024, Sigma) at days 1, 3 and 7. Chondrocytes in each well were lysed with 100 μL sterile distilled DNAse-free H_2_O, and the dissolved solution was collected and transferred into 96-well microtiter plates. 100 μL of 0.1 μg/mL Hoechst 33258 was added to each well and detected with the Microplate Reader (CEMINI XS, Molecular Devices). The fluorescence was recorded at 360 nm excitation and 460 nm emission. Five samples of each group were measured.

### GAG content

Chondrocytes were digested in 0.5 mg/mL proteinase K at 56°C for 12 hours, and were subsequently examined with dimethylmethylene blue (DMMB, 341088, Sigma). The digestive solution was shaken for 30 minutes, then centrifuged at 10000 × g for 10 minutes. The centrifugal sediment was dissolved in a decomplexation solution and absorbance was detected at 630 nm. The GAG content was calculated according to the standard curve by chondroitin sulfate (27042-10G-F, Sigma). Four samples of each group were measured at days 1, 3 and 7.

### Gene expression

Total RNA was extracted and isolated from chondrocytes with Trizol Reagent (206101, New Industry) following the standard protocol, and quantified with Nanodrop spectrophotometer (ND-1000, Thermo). Total RNA (500 ng) was used to perform the reverse transcription reaction with M-MLV reverse transcriptase (C28025, Sigma) and oligo(dT) (FSK-201, TOYOBO) in a PCR thermal cycler (Mycycler, Bio-Rad). Quantitative real-time PCR was performed in the PCR system (Pikoreal 96, Thermo) with RealMasterMix SYBR Green (FP202, Tiangen) following the manufacturer's procedures. The expression of collagen type I (COL I), collagen type II (COL II), collagen type X (COL X), aggrecan (AGG), Sox9, wnt 7a, Lef1, c-jun, cyclin D1, and GAPDH were assessed using PCR with the gene-specific primers listed below: for COL I, 5′- CAG AAC GGC CTC AGG TAC CA-3′ (sense) and 5′- CAG ATC ACG TCA TCG CAC AAC-3′ (antisense); for COL II, 5′- GAG AGG TCT TCC TGG CAA AG-3′ (sense) and 5′- AAG TCC CTG GAA GCC AGAT-3′ (antisense); for COL X, 5′- CAG GTA CCA GAG GTC CCA TC-3′ (sense) and 5′- CAT TGA GGC CCT TAG TTG CT-3′ (antisense); for AGG, 5′- CGA AAC ATC ACC GAG GGT-3′ (sense) and 5′- GCA AAT GTA AAG GGC TCC TC-3′ (antisense); for Sox9 5′-ATC AGT ACC CGC ACC TGC AC-3′ (sense) and 5′-CTT GTA ATC CGG GTG GTC CTT-3′ (antisense); for wnt 7a, 5′- TGC CCG GAC TCT CAT GAA C-3′ (sense) and 5′- GTG TGG TCC AGC AGG TCT TG-3′ (antisense); for Lef1, 5′- CAG TGG ACC CCA AAG GAG AC -3′ (sense) and 5′- CAC AGG TGT GGA TGC AGG AT -3′ (antisense); for c-jun, 5′- CCC CTG TCT CCC ATC GAC ATG-3′ (sense) and 5′- TTG CAA CTG CTG CGT TAG CAT-3′ (antisense); for cyclin D1, 5′- AAC ACG GCT CAC GCT TAC-3′ (sense) and 5′- CCA GAC CCT CAG ACT TGC-3′ (antisense); for GAPDH, 5′- GTC ATC CAT GAC AAC TTC GG-3′ (sense) and 5′- GCC ACA GTT TCC CAG AGG-3′ (antisense). The target genes of each sample were normalized to the values of glyceraldehyde-3-phosphate dehydrogenase (GAPDH) as internal control. Three samples of each group were measured.

### Western blotting

Chondrocytes were lysed by RIPA lysis buffer (R0020, Solarbio) with fresh protease inhibitor of 0.1% phenylmethanesulfonyl fluoride (PMSF, Solarbio). Total cell lysate was boiled after 4 × SDS loading buffer (P1015, Solarbio) was added. Samples were stored at −80° before SDS polyacrylamide gel electrophoresis. Western blotting was carried out according to standard protocol. Rabbit polyclonal antibody against β-catenin (sc-7199, Santa Cruz Biotechnology) and β-actin (4970, Cell Signaling) was combined with HRP-linked antibody of anti-rabbit IgG (7074, Cell Signaling). The complex of the antigen and the antibody was illuminated by chemiluminescence and detected by ChemiDoc XRS + Molecular Imager (BioRad), then quantified by Quantity One image software (BioRad).

### Immunofluorescence

Immunofluorescence was utilized to confirm the location of β-catenin protein. After nsPEF treatment, chondrocytes were fixed with 4% paraformaldehyde for 15 minutes and washed with PBS twice. Cellular permeation was elevated by treatment with 0.5% (v/v) Triton-100, followed by addition of 5% bovine serum albumin (BSA). Chondrocytes were incubated with a primary antibody against β-catenin (sc-7199, Santa Cruz Biotechnology), followed by FITC goat anti-rabbit IgG (0114, Cwbio). The nucleus was stained with DAPI. Relative location between β-catenin and DAPI was observed under the fluorescence microscopy.

### Statistical analysis

Analysis was performed using SPSS V13.0 (SPSS Inc.) one-way ANOVA with the least significant difference (LSD) test (data presented as mean ± s.d). The statistical significance was set at 95% confidence interval, with significance level of p < 0.05.

## Supplementary Material

Supplementary Information Supplementary Information

## Figures and Tables

**Figure 1:**
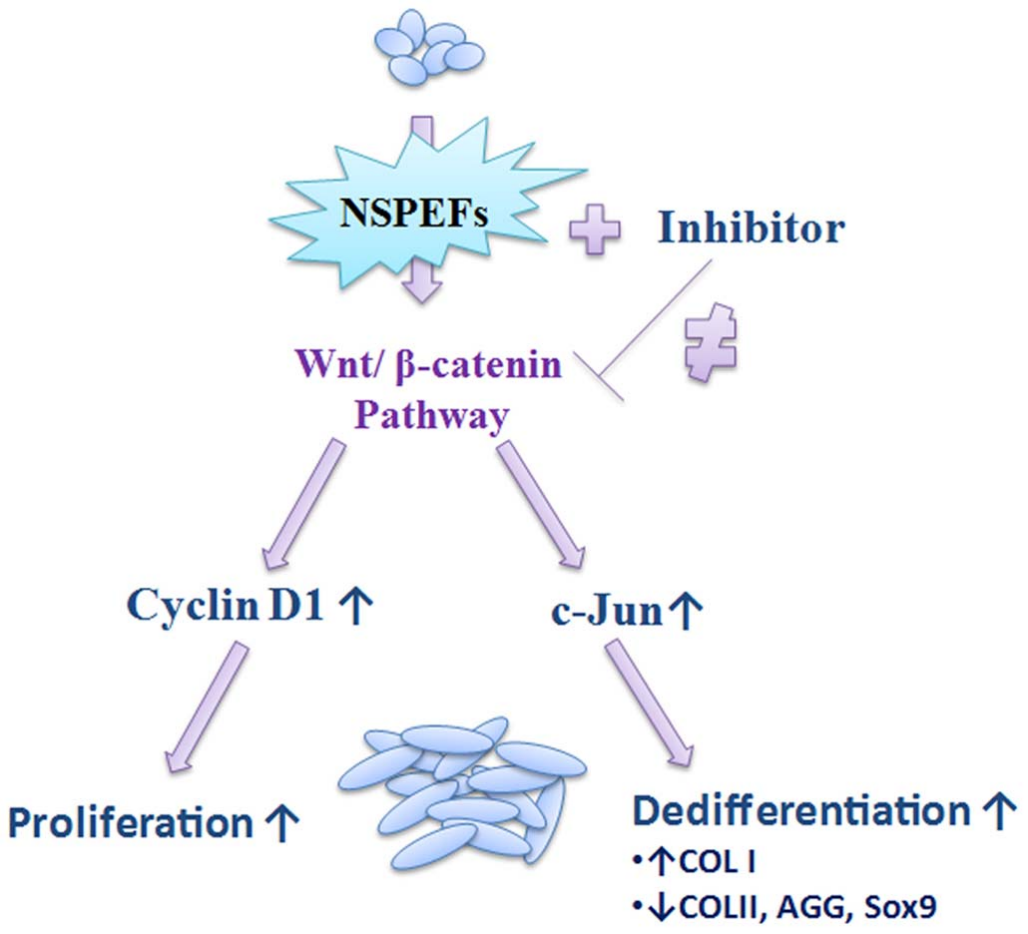
Strategic map exploring the effects of nsPEFs on chondrocytes.
