# Supplementary material for: Nanosecond Pulsed Electric Fields (nsPEFs) Regulate Phenotypes of Chondrocytes through Wnt/β-catenin Signaling Pathway
Source: Sci Rep. 2014 Jul 25;4:5836. doi: 10.1038/srep05836 (PMC5376156; doi:10.1038/srep05836)
Supplement: Supplementary Information [file srep05836-s1.pdf]

Nanosecond Pulsed Electric Fields (nsPEFs) Regulate Phenotypes of Chondrocytes through Wnt/β-catenin Signaling Pathway

Kun Zhang [1], Jinsong Guo [2], Zigang Ge [1, 3, 4, *], Jue Zhang [2, 5, *]

1. Department of Biomedical Engineering, College of Engineering, Peking University, Beijing 100871, China
2. Academy of Advanced Interdisciplinary Studies, Peking University, Beijing 100871, China
3. Center for Biomedical Materials and Tissue Engineering, Academy for Advanced Interdisciplinary Studies, Peking University, Beijing 100871, China
4. Arthritis Clinic and Research Center, Peking University People's Hospital, Beijing, 100871, China
5. College of Engineering, Peking University, Beijing 100871, China

*Correspondence：

Dr. Zigang Ge, Department of Biomedical Engineering, College of Engineering, Peking University, Beijing 100871, China.

Tel.:+8610 62756736; E-mail address: gez@pku.edu.cn

Dr. Jue Zhang, College of Engineering, Peking University, Beijing 100871, China

Tel:+8610 62755036; Email: zhangjue@vip.163.com

Supplementary Information

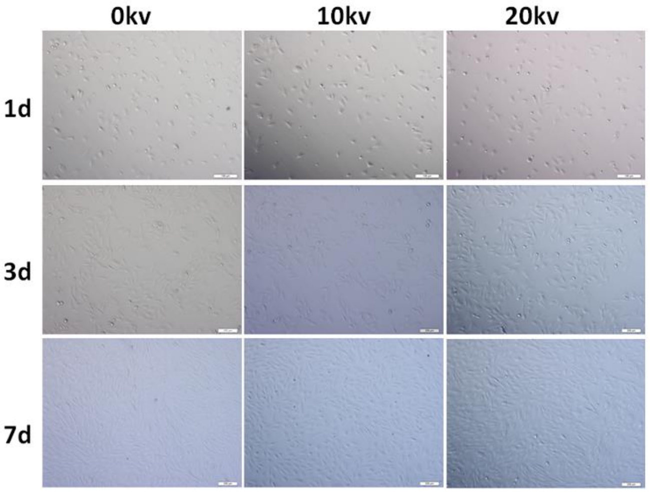

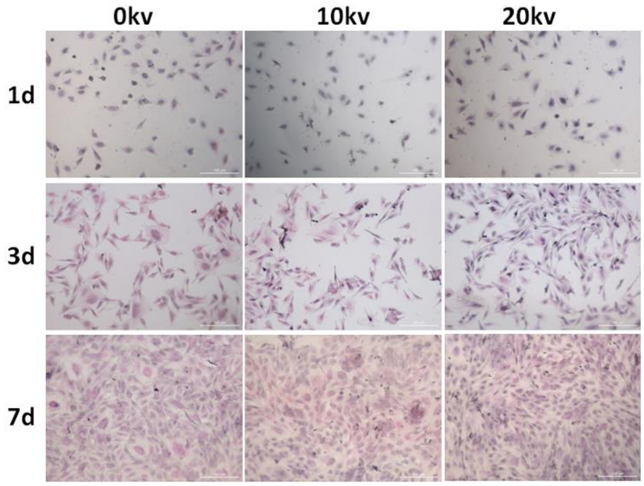

S1a

S1b

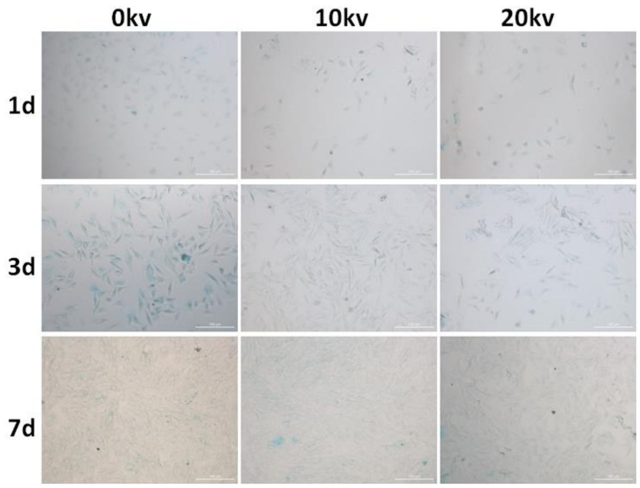

S1c

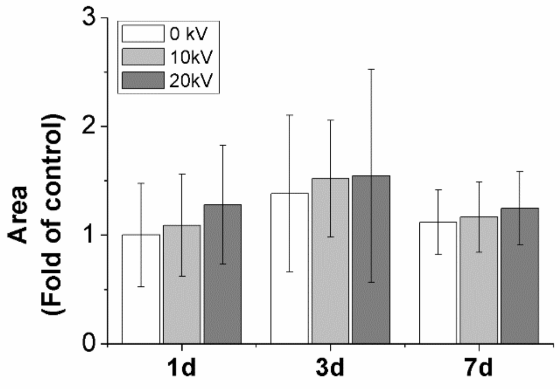

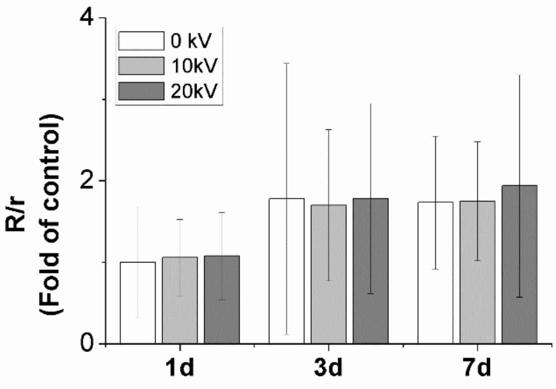

S1d

S1e

Supplementary Figure S1.

Morphology of chondrocytes after nsPEF treatment. (a) Optical microscopy images; (b) H&E staining; (c) Alcian blue staining; (d) Spreading areas of chondrocytes; (e) Rounded phenotype of chondrocytes by ratio of mean maximum (R) and minimum (r) distance between cell’s centroid and contour. Data expressed as mean ± s.d. To evaluate cell morphology, chondrocytes were observed under light microscopy at days 1, 3 and 7. Cellular area and R/r were analyzed using Image-Pro Plus 6.0. Chondrocytes were fixed with 4% paraformaldehyde for 30 minutes and washed with PBS, before haematoxylin & eosin and alcian blue staining.

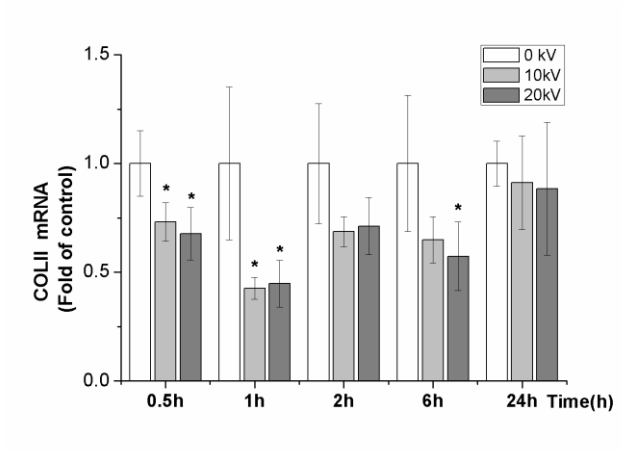

S2a

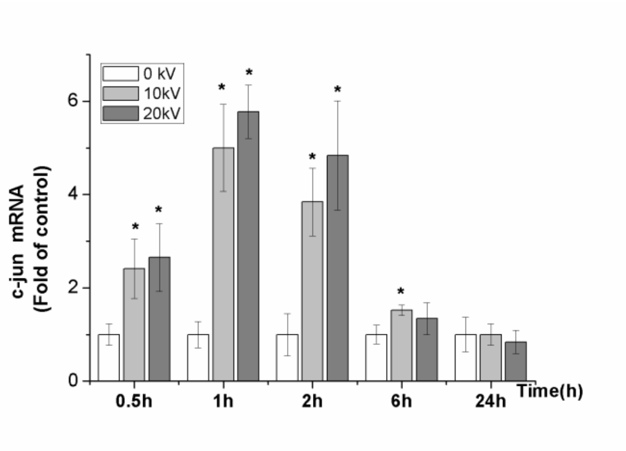

S2b

Supplementary Figure S2

Time course analyses of gene expression of (a) COL II; (b) c-jun at 0.5, 1, 2, 6 and 24 hours after nsPEF treatment. Data expressed as mean ± s.d. *p<0.05.

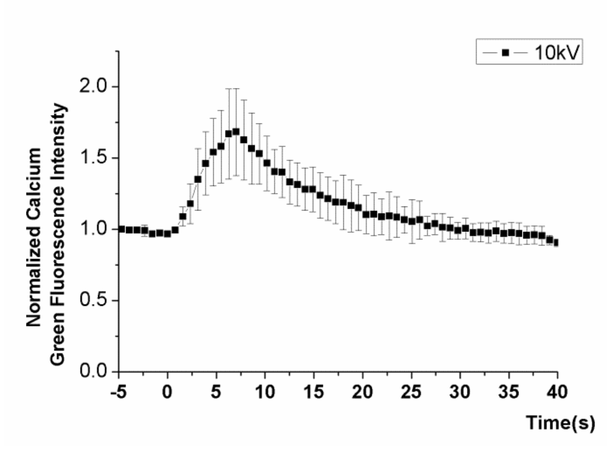

S3a

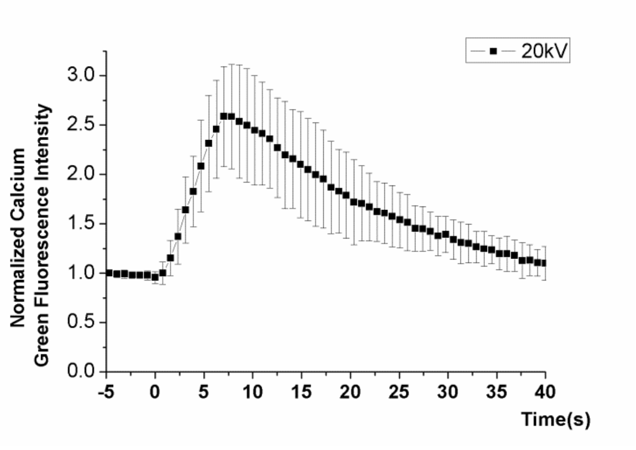

S3b

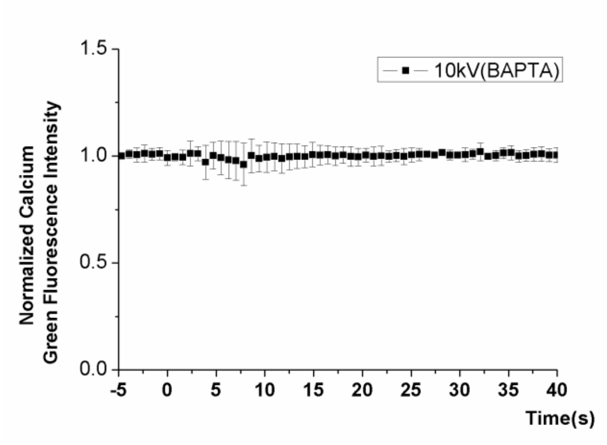

S3c

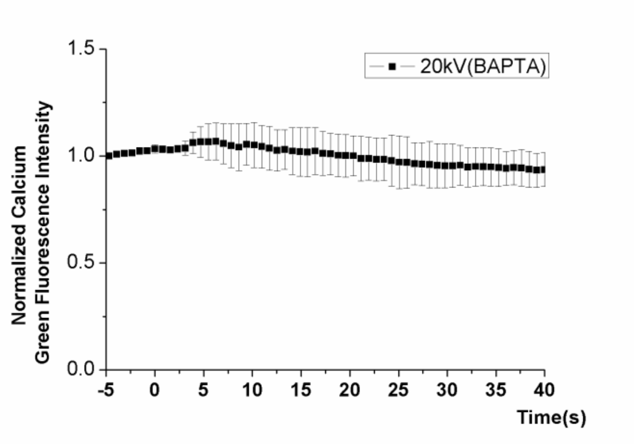

S3d

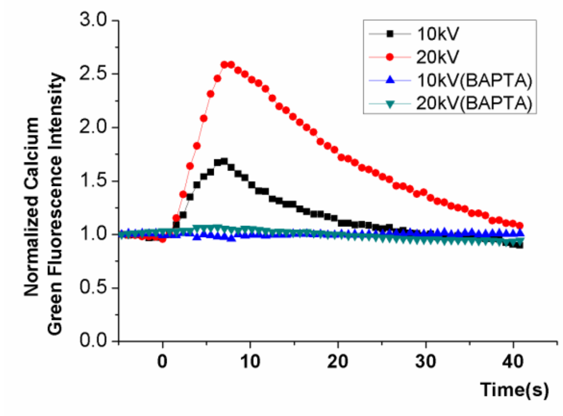

S3e

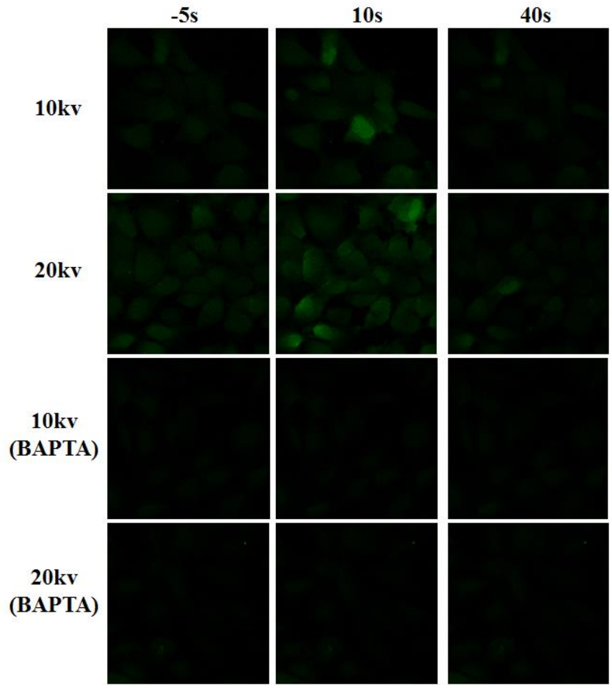

S3f

Supplementary Figure S3

Figure illustrating (a-e) Ca^{2+} fluorescence intensity; (f) confocal images after application of nsPEFs with or without co-treatment with BAPTA-AM. For chemical Ca^{2+} indicator loading, chondrocytes were incubated with 5 μM fluo-4-AM (F14201, Invitrogen) for 10 minutes at room temperature. For Ca^{2+} chelator loading, 15 μM 1,2-bis (2-aminophenoxy) ethane N,N,N',N'-tetraacetic acid (BAPTA)-AM (B1205, Invitrogen) was used to pre-treat chondrocytes for 15 minutes, followed by fluo-4-AM loading for 10 minutes at room temperature, after which nsPEFs were applied. Fluorescent imaging using confocal microscopy (LSM510, Carl Zeiss MicroImaging) was performed with line-scan mode with excitation at 488 nm and emission at 490–570 nm.

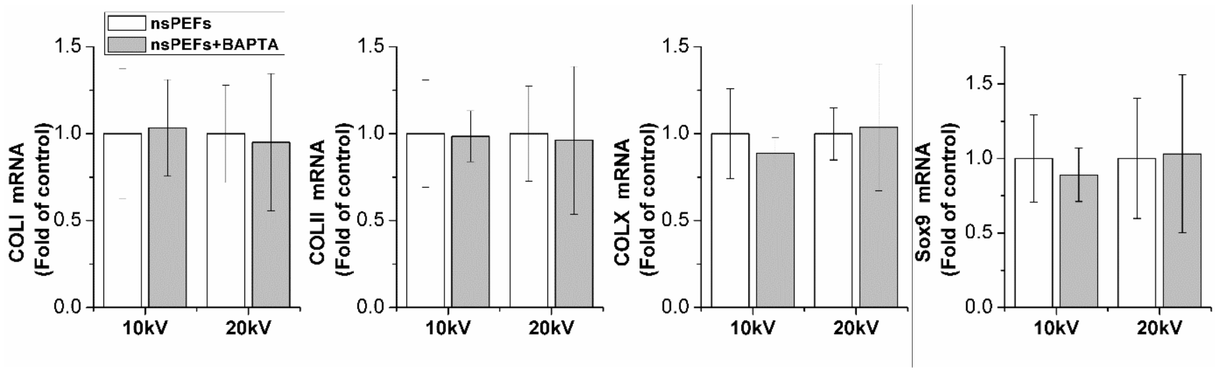

S4a S4b S4c S4d

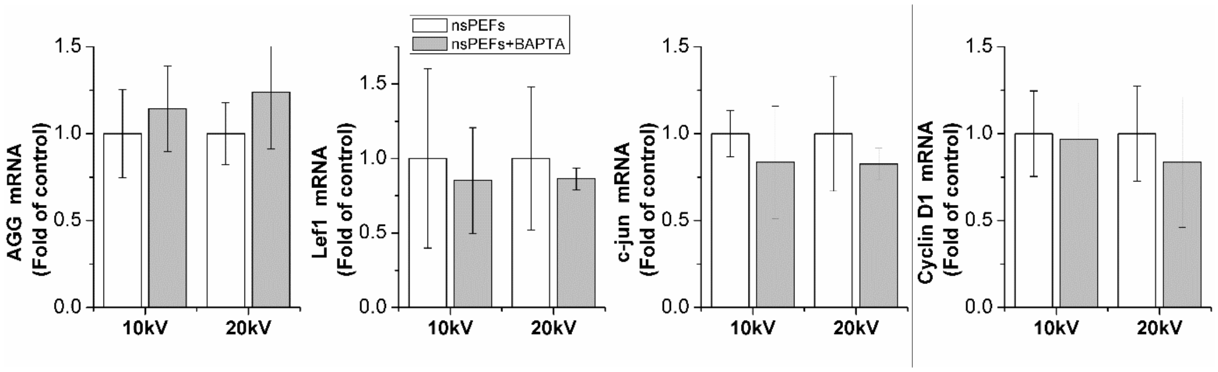

S4e S4f S4g S4h

Supplementary Figure S4

Gene expression after nsPEF treatment compared to co-treatment with BAPTA-AM. (a) COL I; (b) COL II; (c) COL X; (d) Sox9; (e) AGG; (f) LEF1; (g) c-jun; (h) cyclin D1. Data expressed as mean ± s.d.

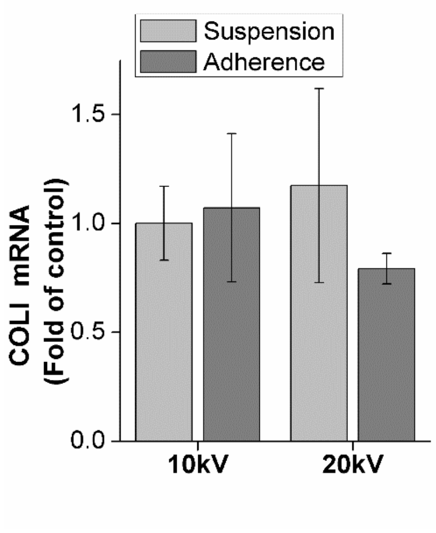

S5a

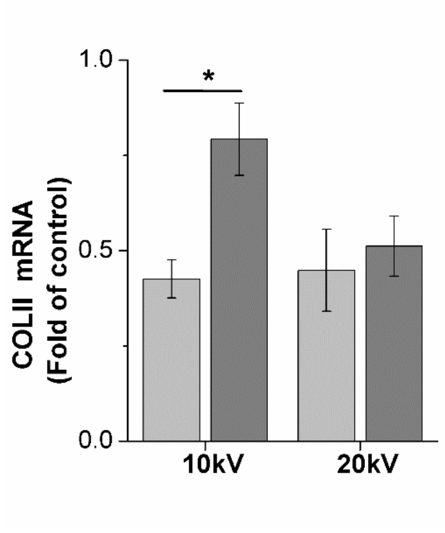

S5b

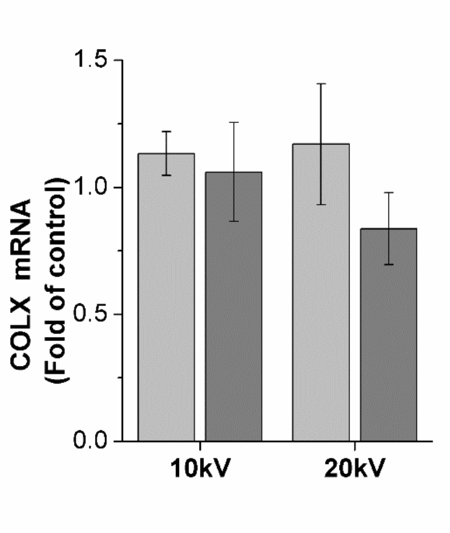

S5c

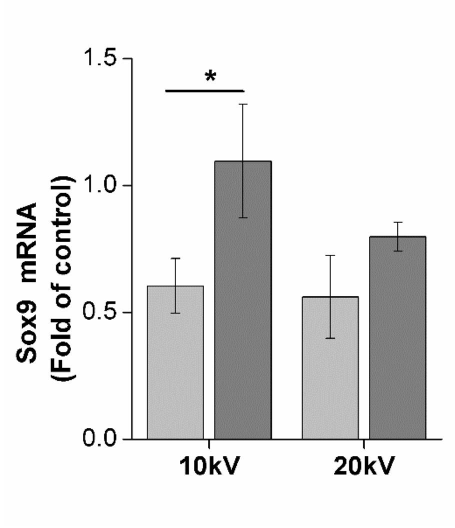

S5d

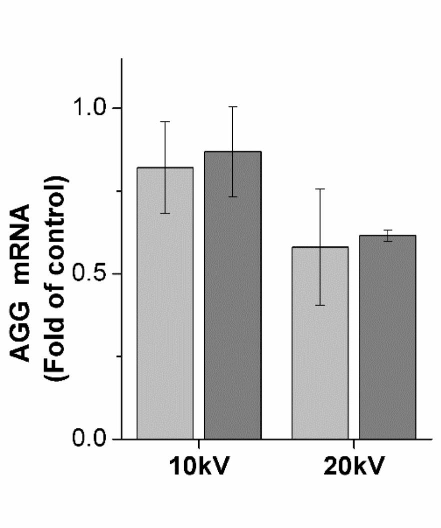

S5e

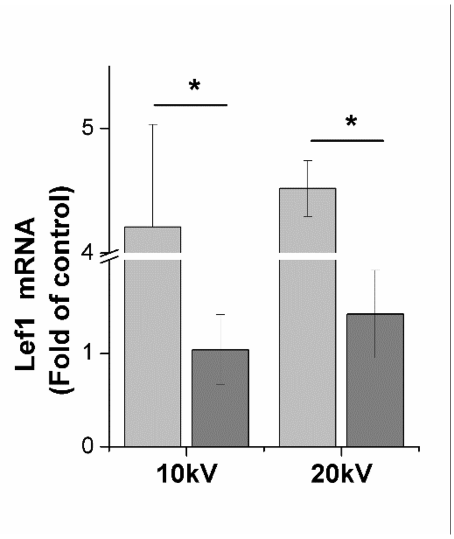

S5f

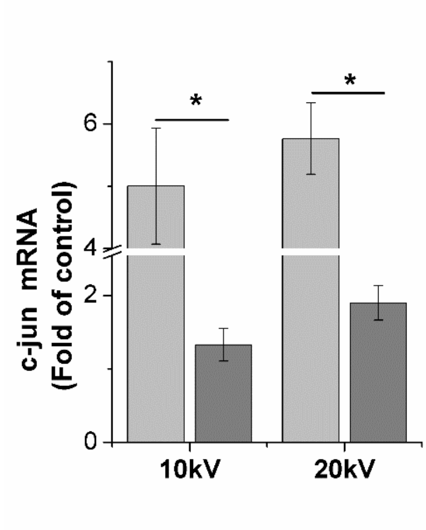

S5g

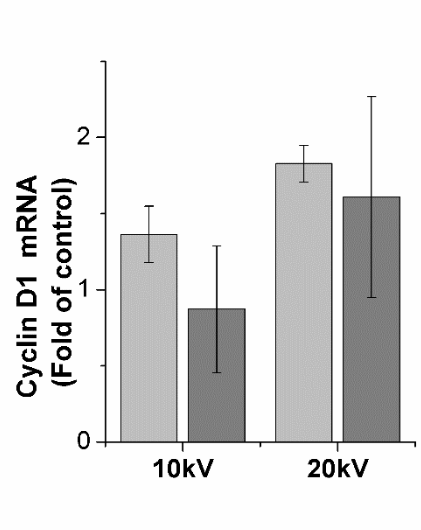

S5h

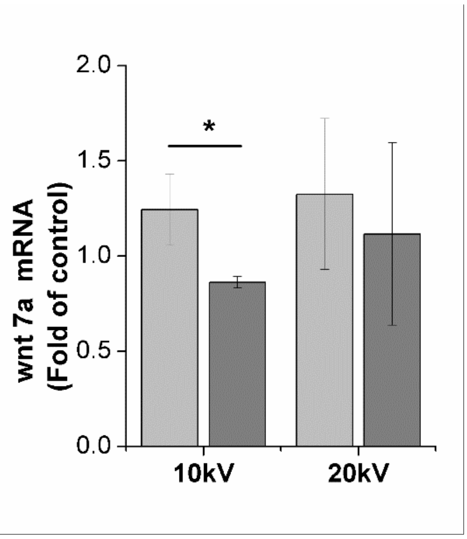

S5i

Supplementary Figure S5

Gene expression of chondrocytes in suspended and adherent states compared with untreated chondrocytes at 1 hour after 10 kV/cm or 20 kV/cm nsPEF treatment. (a) COL I; (b) COL II; (c) COL X; (d) Sox9; (e) AGG; (f) LEF1; (g) c-jun; (h) cyclin D1; (i) wnt7a. Data expressed as mean $\pm$ s.d. $*p<0.05$.
